# Automated Classification System Based on YOLO Architecture for Body Condition Score in Dairy Cows

**DOI:** 10.3390/vetsci11090399

**Published:** 2024-09-01

**Authors:** Emre Dandıl, Kerim Kürşat Çevik, Mustafa Boğa

**Affiliations:** 1 Department of Computer Engineering, Faculty of Engineering, Bilecik Şeyh Edebali University, Bilecik 11230, Turkey; 2 Department of Management Information Systems, Faculty of Applied Sciences, Akdeniz University, Antalya 07070, Turkey; 3 Department of Food Processing, Bor Vocational School, Nigde Ömer Halisdemir University, Nigde 51240, Turkey; mboga@ohu.edu.tr

**Keywords:** dairy cows, body condition score, automatic classification, deep learning, YOLOv8

## Abstract

**Simple Summary:**

This study proposes an automatic classification system for determining body condition score in dairy cows using a deep learning architecture. An original dataset was created by categorizing images of different breeds from different farms into five body condition score classes: Emaciated, Poor, Good, Fat, and Obese. In the experimental analysis, the proposed deep learning model accurately classified 102 out of 126 cow images in the test set, achieving an average accuracy of 0.81 for all classes in Holstein and Simmental cows and an average area under the precision–recall curve of 0.87. The proposed body condition score classification system can help to accurately monitor rapid declines in body condition in dairy cows and serve as a tool for production decision-makers to reduce negative energy balance during early lactation.

**Abstract:**

Body condition score (BCS) is a common tool used to assess the welfare of dairy cows and is based on scoring animals according to their external appearance. If the BCS of dairy cows deviates from the required value, it can lead to diseases caused by metabolic problems in the animal, increased medication costs, low productivity, and even the loss of dairy cows. BCS scores for dairy cows on farms are mostly determined by observation based on expert knowledge and experience. This study proposes an automatic classification system for BCS determination in dairy cows using the YOLOv8x deep learning architecture. In this study, firstly, an original dataset was prepared by dividing the BCS scale into five different classes of Emaciated, Poor, Good, Fat, and Obese for images of Holstein and Simmental cow breeds collected from different farms. In the experimental analyses performed on the dataset prepared in this study, the BCS values of 102 out of a total of 126 cow images in the test set were correctly classified using the proposed YOLOv8x deep learning architecture. Furthermore, an average accuracy of 0.81 was achieved for all BCS classes in Holstein and Simmental cows. In addition, the average area under the precision–recall curve was 0.87. In conclusion, the BCS classification system for dairy cows proposed in this study may allow for the accurate observation of animals with rapid declines in body condition. In addition, the BCS classification system can be used as a tool for production decision-makers in early lactation to reduce the negative energy balance.

## 1. Introduction

The welfare of dairy cows is assessed by taking into account their physiological and clinical status, as well as their productive performance [1]. Improving on-farm care and nutrition plays an important role in preventing metabolic diseases in dairy cows [2]. The occurrence of metabolic diseases in dairy cows can lead to low productivity and be detrimental to farms in various ways, such as increased veterinary costs and reduced performance [3]. On dairy farms, animals are grouped according to BCS and their care and feeding is carried out at specific times [4]. BCS, which is an indirect measure of subcutaneous fat content in dairy cows, is widely used in farm animal management [5]. The use of BCS is effective for the assessment of nutritional status, particularly in dairy cows [6]. 

Dairy farms play a crucial role in the agricultural sector, raising cows for milk production. Effective farm management practices are essential to ensure the health and well-being of these animals, ultimately impacting the quality and sustainability of milk production. There are many practices for managing animals on farms, such as housing, feeding, watering, breeding and reproduction, and care [7,8]. On farms, it is not desirable for animals to be either too thin or too fat. On dairy farms, the lactation cycle needs to be closely monitored at specific times [9]. As BCS is an important indicator of whether the needs of dairy cows are being met, determining an appropriate BCS value can prevent unnecessary feed consumption and increase yield [10]. Furthermore, BCS is an important indicator because it is used as the primary method, based on subjective analysis, to determine energy balance and reserves in dairy cows. Regular BCS checks in dairy cows make it possible to increase milk yield, prevent metabolic diseases, and meet the cows’ needs. In addition, grouping with BCS is very important to avoid overfeeding or underfeeding dairy cows. In this sense, it is also possible that a regular allocation of dairy cows belonging to each BCS group can increase farm profits.

Traditionally, BCS determination of dairy cows is carried out by trained animal caretakers or specialists by visual or tactile assessment of the lumbar and sacral regions [5]. BCS is assessed using the basic bone structures for fat cover in cows [11]. The relationship between subcutaneous fat thickness in the dorsal, lumbar, and coccygeal regions of dairy cows and pelvic bony prominences is determined visually or by palpation using the BCS grouping method [12]. Most BCS systems use a scoring approach from 1.0 to 5.0 in quarter- or half-point increments [13,14]. The BCS for dairy and beef cows can be measured using different scales. Sometimes a five-point scale [15] is used for BCS, while sometimes an eight-point scale [16] is used for dairy or beef cows. In addition, a nine-point scale [5,6,17] is often used in certain management practices for dairy or beef cows and a ten-point scale [18] is utilized in various settings or research. These scales can also vary in increments, with some using 0.5 increments and others using 0.25 increments, to provide a more precise assessment of the BCS.

In BCS, the fatness or leanness of dairy cow is scored on a scale from 1.0 to 5.0 [19], with a score of 1.0 representing very lean cows and a score of 5.0 representing very fat cows [14,15,20]. Furthermore, BCS = 1.0 in lean animals represents cows that are characterized as emaciated and indicates that the herd may have metabolic problems from reproduction to milk production [21,22]. A BCS of 1.0 or close to 1.0 in dairy cows can lead to low milk yield after calving and economic losses manifested by reproductive problems in animals [9]. On the other hand, fat animals may have some problems such as labor difficulties and metabolic disorders [23]. High condition scores can occur when fat animals enter labor. In addition to reproductive problems, fat animals often have labor problems. After calving in dairy cows, body condition scores decrease to BCS = 2.5 in high-yielding animals and can increase to BCS = 4.0 in the period just before calving. In addition, conditions above BCS = 4.0 are generally not favored on farms, as cows may have metabolic problems after parturition if BCS is above 4.0. Accordingly, metabolic problems such as inadequate and unbalanced feeding and high negative energy balance may occur. In addition, the late diagnosis of diseases that can occur in dairy cows on farms can lead to economic losses and the loss of the animal from the herd. The intention is to ensure that the animal goes into labor with a fat deposit that is not excessive. Therefore, it is very important to accurately determine BCS in dairy cows.

Various methods are used by experienced specialists for farm applications of dairy cows. Additionally, technologies such as robotic milking systems, pedometers, and mobility monitoring are used to maintain hygiene and animal health on farms [24]. The number of on-farm solution applications for early reproductive symptoms in dairy cattle is also increasing [25]. However, on some farms with small numbers of animals and no access to expensive solutions, the inability to make correct BCS groupings in dairy cows and the lack of computer-aided tools to assist specialists means that diseases are diagnosed late and treatment cannot be administered in a timely manner. In fact, less than 5% of dairy farmers are estimated to regularly use BCS grouping [5]. On dairy farms, BCS groupings performed by experts based on visual inspection involve subjective judgments and may result in incorrect BCS values [26]. Therefore, it is very important to have systems that can be used as a tool by experts due to BCS grouping on the farm and that allow early action to be taken with correct results.

Many studies have been proposed for BCS assignment in animals [27,28,29,30]. Although some of these studies are designed to classify BCS in dairy cows, there are also studies on the detection of BCS in other animals. When evaluating the studies proposed for BCS detection by processing cow images, it can be seen that most studies are proposed as automatic BCS estimation. There are approaches that focus on the use of digital images to measure BCS in dairy cows for use in farm management. In one of these approaches, it has been proposed to use video image analysis to measure conformation and body size traits in dairy cows [31]. There are also approaches that highlight the use of digital images that can be provided remotely to farm advisors for the assessment of BCS in the nutritional management of dairy cows [14]. These approaches show similar results between image-based BCS scoring and live cow scoring by an experienced expert. On the other hand, Halachmi et al. [32] presented an automatic method for BCS estimation in dairy cattle using thermal images. In the study, the body shapes of the animals were taken into account using advanced image processing models. In another study, Spoliansky et al. [33] developed an automated BCS approach for dairy cows using data from a 3D Kinect camera with image processing algorithms. Three-dimensional feature extraction and multiple body regions were used for automated BCS detection in dairy cows by Song et al. [34]. In their study, Martins et al. [35] used 3D cameras and manual body measurements to estimate BCS and body weight in dairy cows.

In recent years, several studies have explored deep learning techniques for automated BCS classification in dairy cows. Rodriguez Alvarez et al. [36,37] employed convolutional neural networks (CNNs) for BCS estimation, with one study incorporating ensemble modeling for improved accuracy [37]. Huang et al. [8] utilized a single-shot multibox detector (SSD) for BCS detection, while Li et al. [38] combined YOLOv2 with CNN for BCS prediction. Çevik [39] further developed a real-time classification system based on pre-trained deep learning architectures and a mobile application for dairy farmers. Nagy et al. [40] investigated BCS classification in dairy cows using a deep learning approach, exploring the impact of annotation and score classes. He et al. [41] proposed a YOLOX-based system for BCS classification, introducing improvements to the base architecture and comparing its performance with that of existing methods. These studies demonstrate the increasing adoption of deep learning for automated BCS classification in dairy cows. 

For the development of automated BCS classification systems, recent advances in deep learning offer promising opportunities. While several studies have explored deep learning approaches for BCS classification, there exist limitations that our work addresses. Existing studies have primarily focused on architectures like CNN or adaptations of these architectures. Our work contributes to this growing field by investigating the application of the YOLOv8x architecture for BCS classification, with a focus on its efficiency and potential for on-farm applications. YOLOv8x offers object detection and classification capabilities, potentially leading to improved efficiency compared with traditional CNN-based approaches. In addition, the diversity and representativeness of datasets used in previous studies can vary. Our work utilizes a well-annotated and diverse dataset specifically focusing on Holstein and Simmental crossbred dairy cows. This dataset captures variations in BCS across different farm environments, potentially leading to a more generalizable model compared with studies using limited datasets or specific breeds.

The contribution of this study is the development and evaluation of an automated classification system using the YOLOv8x deep learning architecture to accurately determine the BCS of dairy cows of Holstein and Simmental breeds. In this study, a classification system based on YOLOv8x deep learning architecture is proposed for the automated detection and classification of BCS from images containing key areas, such as the backbone, pins, hips, and tailhead, of dairy cows of different breeds. The proposed system would enable the early identification of weak or obese animals with true assignment of BCS on the farm and the provision of the necessary care and feeding. The highlights of the proposed study can be explained as follows:

- This study produced an original large-scale dataset of images collected from 20 different farms for different breeds of cows, including Simmental and Holstein;
- The BCS scale in cows was divided into five different classes of Emaciated, Poor, Good, Fat, and Obese;
- Hyperparameter optimization in the single-stage structure of the YOLOv8x deep learning architecture for BCS classification in dairy cows is proposed for the first time to the best of our knowledge;
- The performance of the proposed work is compared with that of other previously proposed state-of-the-art methods and it is verified that it contributes in terms of accuracy.

## 2. Materials and Methods

In this study, a comprehensive dataset was created by collecting images of dairy cows from various farms, ensuring a diverse representation of different breeds and environments. Each cow image was annotated with a BCS by experts, categorizing the cows into five classes: Emaciated, Poor, Good, Fat, and Obese. The annotated images were standardized by resizing them to a uniform resolution suitable for the YOLOv8x model. The YOLOv8x deep learning model, chosen for its efficiency in object detection and classification, was trained using these images. Key hyperparameters for YOLOv8x, like learning rate, batch size, and the number of epochs, were optimized through experimentation. Comprehensive assessment was performed for the model’s ability to accurately classify the BCS of dairy cows, highlighting its strengths and areas for improvement.

### 2.1. Dataset

In this study, the first step was to obtain images for BCS. For this purpose, 20 livestock farms with at least 100 milking cows were selected for data collection. A dataset was created by collecting back images of Holstein and Simmental crossbred dairy cows. Cow images for the dataset were obtained from farms in the Nigde Region of Türkiye between 1 March and 31 May 2023. The images in the dataset were collected by 5 different zootechnicians who were experts in their field at graduate and undergraduate levels. Each cow was photographed in a similar position and at similar angles within a farm. When collecting images of the cows on the farms, care was taken to ensure that the cows were in the milking period, which was an average of 300 days in the production cycle. Cows were imaged three more times at 1-month intervals on the same farms after the images were captured at each stage from birth. Poorly captured cow images were pre-processed and unacceptable images were deleted. Afterward, the actual BCS scores (ground truth) of the dairy cow images from different farms were grouped according to the 5 different BCS scales as Emaciated (BSC = 2.0), Poor (BCS = 2.5), Good (BCS = 3.5), Fat (BCS = 4.0), and Obese (BCS = 4.5) by an expert zootechnician working at Niğde Ömer Halisdemir University. Staufenbiel’s study [6,17] was used as a reference for the BCS classification and class labels. In this study, the BCS classification of cows was carried out based on the expert’s experience after a detailed examination of the images obtained. In addition, the BCS groups of dairy cattle were determined with an accuracy of 0.5. However, in the farms where cattle images were collected, no cattle images were marked by the expert for BCS = 1.0 and BCS = 1.5 for cattle images. Similarly, in the farms where images were collected in the dataset, no sub-grouping was applied as there were no cattle images for BCS = 5.0. In addition, as the cattle images for BCS = 3.0 and BCS = 3.5 were very similar, the expert identified all cattle in this group as BCS = 3.5. The images were collected by zootechnicians using several different mobile devices based on Android and iOS operating systems to show the bone ridges in the pelvic region of the animals. 

**Figure 1:**
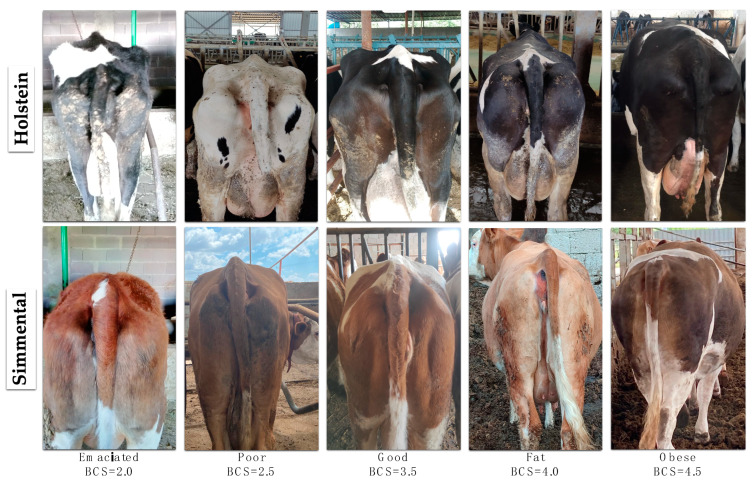
Sample back images of Holstein and Simmental cows in the dataset for the BCS classes of Emaciated, Poor, Good, Fat, and Obese.

In this study, we classified dairy cows into five distinct BCS categories: Emaciated, Poor, Good, Fat, and Obese. The Emaciated class represents animals that are not desired in the herd due to their extremely low body condition. The Poor class includes cows that are physically compromised, but can be treated and managed with appropriate interventions. The Good class comprises animals that are in optimal condition, monitored from birth and maintained in an ideal state. The Fat class consists of cows that are generally healthy, but require dietary adjustments to prevent potential metabolic issues. The Obese class includes cows that are excessively overweight and thus undesirable in the herd. Monitoring visual signs of high or low BCS is crucial for maintaining herd health. High or low BCS necessitates nutritional adjustments on the farm to ensure the well-being of the animals. Ideally, dairy cows should have a Good class (BCS = 3.5) at the time of calving. The first step in our study was to identify and collect images of cows with a Good class (BCS = 3.5). Subsequently, experts collected images of cows with varying BCS, and the animals were classified according to their condition scores. Figure 1 presents sample images of Holstein and Simmental cows classified as Emaciated (BCS = 2.0), Poor (BCS = 2.5), Good (BCS = 3.5), Fat (BCS = 4.0), and Obese (BCS = 4.5) from the dataset created for this study. By systematically categorizing cows based on BCS and monitoring these scores, we can implement targeted nutritional and management strategies to maintain optimal herd health and productivity.

**Figure 2:**
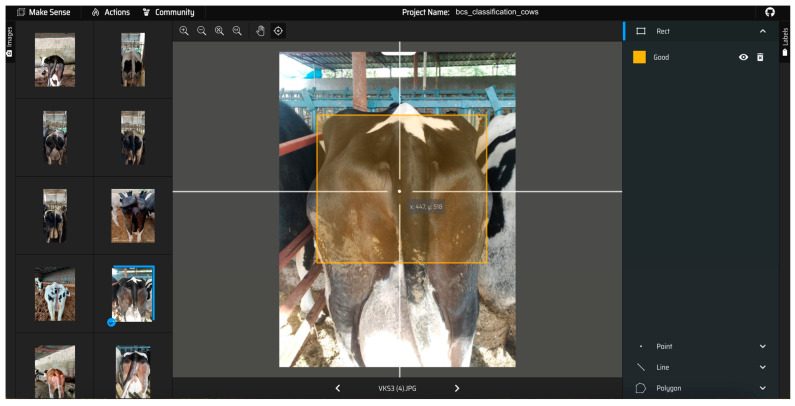
In the proposed study, using the Make Sense [42] application, preparing ground truth class labels and regions of interest on a cow’s image in a YOLO-compliant manner for BCS classification.

The dataset created as part of this study consisted of a total of 1270 images of cows. While 925 of these images were of Holstein cows, the remaining 345 images were of Simmental cows. For the dataset generated in this study, ground truth class labels (annotations) were manually generated in a YOLO-compatible manner using the application Make Sense [42] for 5 different BCS values, as shown in Figure 2. The generated labels were created by consensus of all zootechnical experts. In the BCS scores for dairy cows, Holstein breeds were well-balanced in each BCS group, whereas Simmental cows were generally in the higher scoring range of the BCS evaluation due to their genetically larger appearance. During data collection, all experts independently labeled all 1270 cows. Prior to the ground truth phase, the experts were trained by a zootechnician to apply the manual BCS protocol.

### 2.2. YOLOv8x

**Figure 3:**
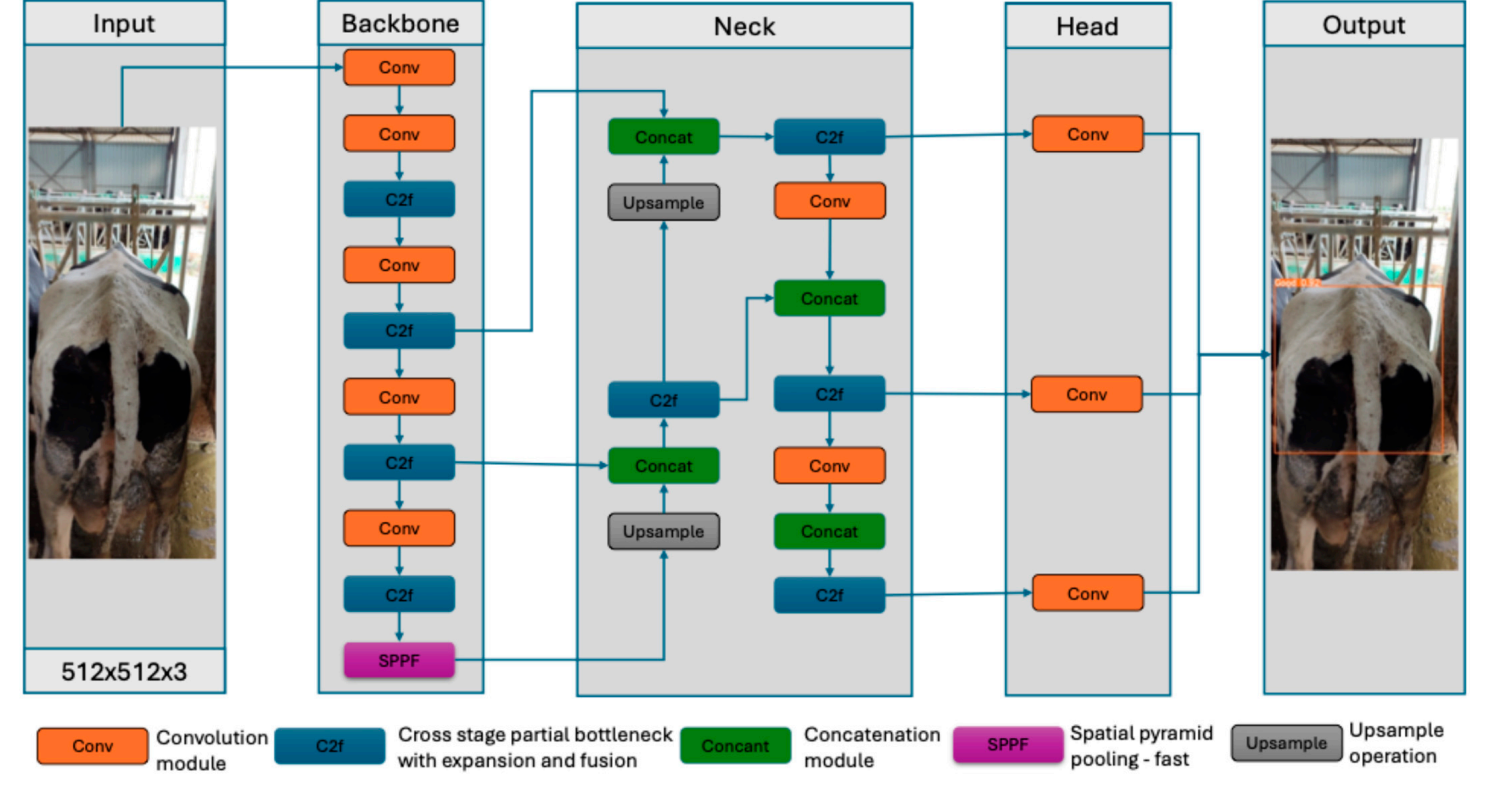
The architecture of the YOLOv8x deep learning network used for BCS classification in dairy cows.

YOLOv8, one of the most advanced models in the YOLO series, is very effective in terms of accuracy and speed in single-stage object recognition. In addition, although this YOLO model has many frameworks, YOLOv8x is ahead of the others in terms of depth and breadth. In particular, YOLOv8x has the ability to achieve higher scores in terms of classification accuracy [43]. YOLOv8x consists of four main distinct components, namely Input, Backbone, Neck, and Head [44]. The Input module consists of three subcomponents: mosaic data augmentation, adaptive anchor box computation, and adaptive image scaling. Backbone mainly uses C2f and GIS modules to extract features from images. The neck part of YOLOv8x is responsible for combining features from different layers and detecting objects of different sizes. Finally, the head part consists of convolutional layers, pooling layers, and fully connected layers, which are necessary for object detection, classification, and output. Figure 3 shows the YOLOv8x deep learning architecture proposed in this study for BCS detection in dairy cows.

### 2.3. Infrastructure of Hardware and Software

The YOLOv8x model was trained and optimized on a portion of the dataset, and its performance was evaluated on a separate test set containing unseen images. By analyzing these results, we gain valuable insights into the strengths and weaknesses of the YOLOv8x model for automated BCS classification. As part of the study, the characteristics of the computer and hardware structures used for the experimental studies are presented in Table 1. Ultralytics YOLOv8.0.176 distribution was used for training the model trained on the local machine. Python version 3.11.6, Torch version 2.0.1 + cu117, and CUDA version 11.0 were used. 

The optimization of the hyperparameters used to train the YOLOv8x network is shown in Table 2. The optimal epoch for training the network was determined to be 300. The batch size of 16 in YOLOv8x provides a practical starting point for training. The optimizer plays a crucial role in how the model learns and updates its weights during training. Choosing the right optimizer has a significant impact on factors such as convergence speed, training stability, and generalizability. Since stochastic gradient descent (SGD) performs well in various optimization tasks, making it a reasonable starting point for YOLOv8 training, SGD was chosen as the optimizer. In YOLOv8x training, the value of 4 for the mask ratio plays a crucial role in balancing efficiency and accuracy when generating segmentation masks for objects. The optimal values for the other hyperparameters in YOLOv8x, such as learning rate, IoU, momentum, mosaic, dropout, and weight decay, are also given in Table 2.

**Figure 4:**
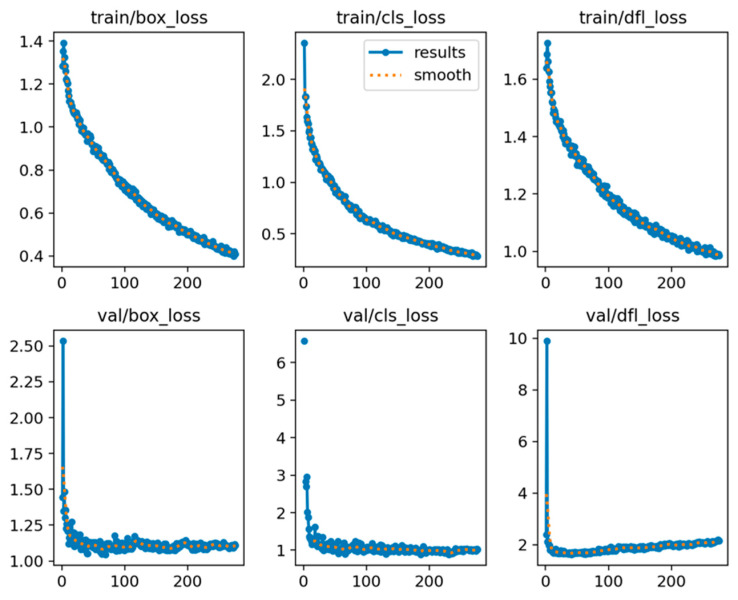
Training loss and validation loss curves resulting from training the YOLOv8x network for 300 epochs.

The training loss and validation loss curves of the YOLOv8x network proposed for BCS classification in dairy cows, whose hyperparameters were optimized as a result of training for 300 epochs, are shown in Figure 4. Box loss (box_loss) measures the difference between the predicted bounding boxes by the model and the actual (ground truth) bounding boxes for the objects present in the image. Accurate bounding boxes are crucial for object detection, and minimizing this loss ensures that the model learns to predict boxes that tightly enclose the target objects. Class loss (cls_loss) measures the YOLOv8x model’s ability to correctly classify the objects present in each image. Minimizing this loss ensures that the model learns to distinguish different object classes effectively. Distribution focal loss (dfl_loss) is a variant of the focal loss function specifically designed to address the issue of class imbalance in object detection datasets. This imbalance can lead the model to prioritize learning the frequent classes. These loss curves in training and validation show that the training of the YOLOv8x network was successful and the network learned enough for classification.

## 3. Results

The distribution of images in the dataset according to BCS scale and class labeling is shown in Table 3. In this study, 90% (1144) of the total 1270 images in the dataset were used for training and the remaining 10% (126) were used for testing. The selection of training and test images from the dataset was completely randomized. For Simmental cows, there were 306 images in the training set and 39 images in the test set, giving a total of 345 images. On the other hand, for Holstein breeds, there were 838 and 87 images in the training and test sets, respectively, giving a total of 925 images.

Table 4 shows the confusion matrix resulting from the classification of 126 Holstein and Simmental cow images for five different BCS classes in the test set in the dataset prepared in this study with the proposed YOLOv8x deep learning architecture. It can be seen that the BCS classification of all cows’ images in the Emaciated class was performed correctly. On the other hand, 17 out of 25 images in the Poor class were correctly classified, while 7 of these images were misclassified in the Good class and 1 in the Fat class. On the other hand, out of a total of 49 cow images in the Good class, 44 cows were correctly classified, while the BCS value of 4 images was incorrectly classified in the Fat class and 1 image was incorrectly classified in the Poor class. In the Fat class with 35 test images, the BCS of 27 cows was correctly determined, while the BCS of the remaining 8 cows was incorrectly classified as Good. Finally, the BCS value of 6 out of 9 test images in the Obese class was correctly classified, whereas 2 of the remaining 3 images in the Fat class and 1 in the Good class were incorrectly classified. As a result, the BCS values of 102 out of a total of 126 cow images in the test set were correctly classified using the proposed YOLOv8x deep learning architecture. Furthermore, the accuracy scores achieved with YOLOv8x for each class are presented in Table 5. On the test set, an average accuracy score of 0.81 was obtained for all classes.

**Figure 5:**
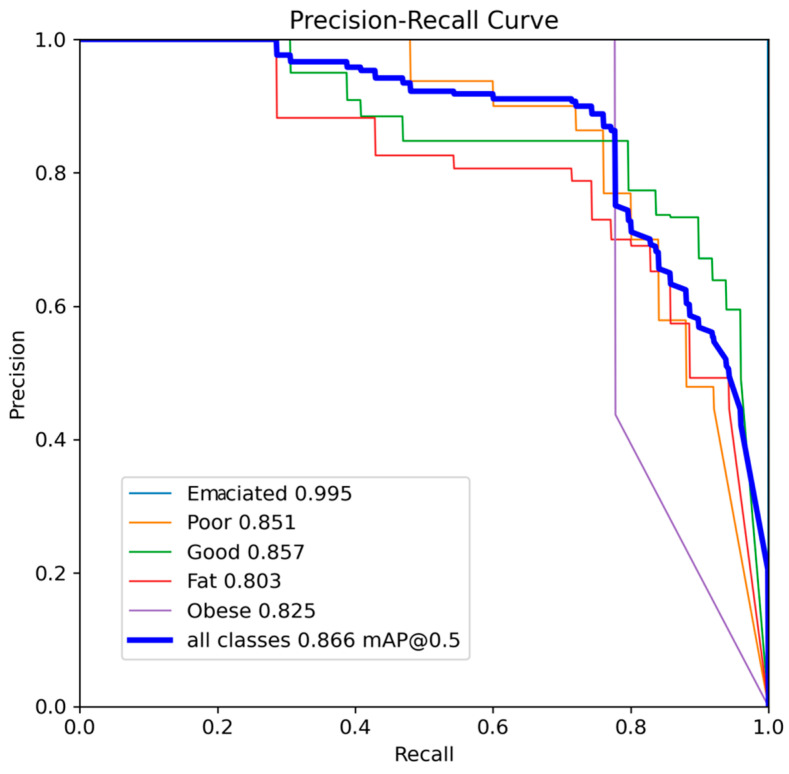
Precision–recall curve in BCS classification for each class.

In the experimental studies conducted as part of this study, the average precision–recall curves (PRCs) achieved for each class and all classes in the BCS classification of cow images in the test set are shown in Figure 5. Precision and recall metrics are very useful for measuring prediction results when classes are unbalanced. The PRC shows the relationship between precision and recall for different threshold values, and the larger the area under this curve, the higher the recall and precision results. In this study, in the BCS classification of dairy cows, the highest success was achieved in the Emaciated class with 0.995 in terms of the size of the area under the PRC curve. The lowest performance was obtained in the Fat class, with 0.803 in terms of the area under the PRC curve. On the other hand, when the threshold for mAP was set at 0.5 for BCS in all classes, the area under the PRC curve was 0.866.

This study also evaluated the effect of Simmental and Holstein cows in the dataset separately for BCS classification. Table 6 shows the confusion matrix that indicates the classification result for each BCS class for a total of 39 Simmental cow images in the test set. Out of the 39 Simmental cow images, 31 images were correctly classified in terms of BCS, while 8 images were incorrectly classified in terms of BCS. Looking at the misclassifications for BCS in Simmental cow images, 2 Poor-class images were classified as Good class, 3 Good-class images were classified as Fat class, 2 Fat-class images were classified as Good, and 1 Obese-class image was classified as Fat class. In addition, the average accuracy of BCS classification in Simmental cows was 0.80.

Table 7 shows the classification results for each BCS class for a total of 87 Holstein cow images in the test set. Of the 87 Holstein cow images, 71 of the BCS classifications were correct, while 16 images had incorrect BCS classifications. Considering the misclassifications for BCS in Holstein cow images, five images from the Poor class were classified as Good class and one image was classified as Fat class, one image from the Good class was classified as Fat class and one image was classified as Poor class, six images from the Fat class were classified as Good class, and one image from the Obese class was classified as Fat and one image was classified as Good class. In addition, the average accuracy in the BCS classification of Holstein cows was 0.82. When the BCS classification results of Simmental and Holstein cows were evaluated together, a slightly higher classification accuracy was achieved in the BCS determination of Holstein cows.

## 4. Discussion

**Figure 6:**
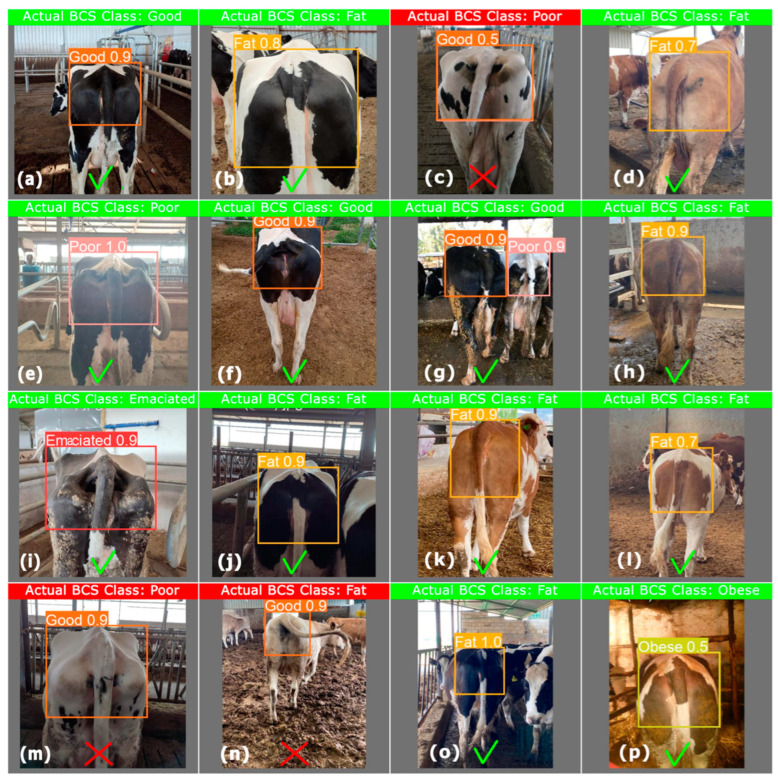
The visual results of BCS classification in dairy cows using YOLOv8x. Correct classification in (**a**,**b**,**d**–**l**,**o**,**p**) as the actual BCS class and predicted BCS class are the same, incorrect classification in (**c**,**m**,**n**) as the actual BCS class and predicted BCS class are not the same.

The results of the BCS classification obtained for some of the Holstein and Simmental cow images in the test set using YOLOv8x in the proposed study are shown in Figure 6, together with the mAP values accepted as the confidence level. While some of the given sample BCS image results were correct, some were classified as incorrect. In these classifications, the threshold for mAP was 0.5 and above. This study did not estimate other intermediate groups for BCS classification, except for five different BCS classes of Emaciated (BSC = 2.0), Poor (BCS = 2.5), Good (BCS = 3.5), Fat (BCS = 4.0), and Obese (BCS = 4.5). With the proposed YOLOv8x, BCS classifications in dairy cows were mostly correct based on the actual BCS classes, while some were incorrectly classified, as shown in Figure 6c–n. Although the actual class in Figure 6c,m was Poor, the proposed method predicted the BCS classification as Good in both images. In Figure 6n, although the actual class was Fat, the prediction result was again Good. Although there were many reasons for the misclassification, the similarity of the class images and the posture of the animal could be seen as the main reasons for the incorrect BCS classification. In addition, as can be seen in Figure 6g, for some images, the proposed YOLOv8x deep learning architecture could predict more than one class label and form a bounding box. In this case, a high-confidence BCS classification was considered.

**Figure A1:**
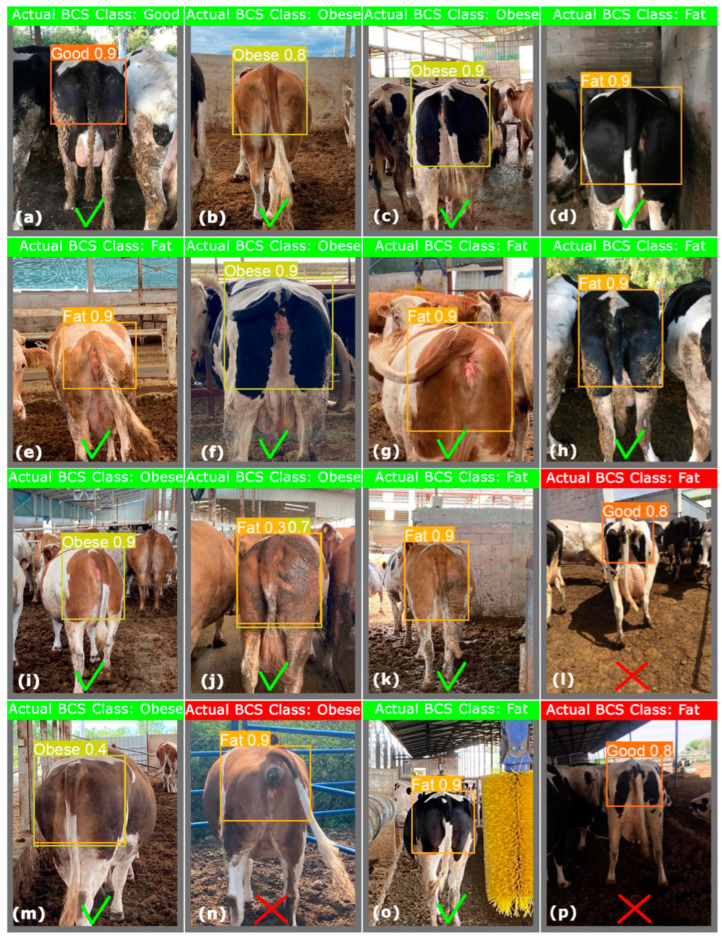
Correct (**a**–**k**,**m**,**o**) and incorrect (**l**,**n**,**p**) classifications in test set images for BCS in dairy cows.

**Figure A2:**
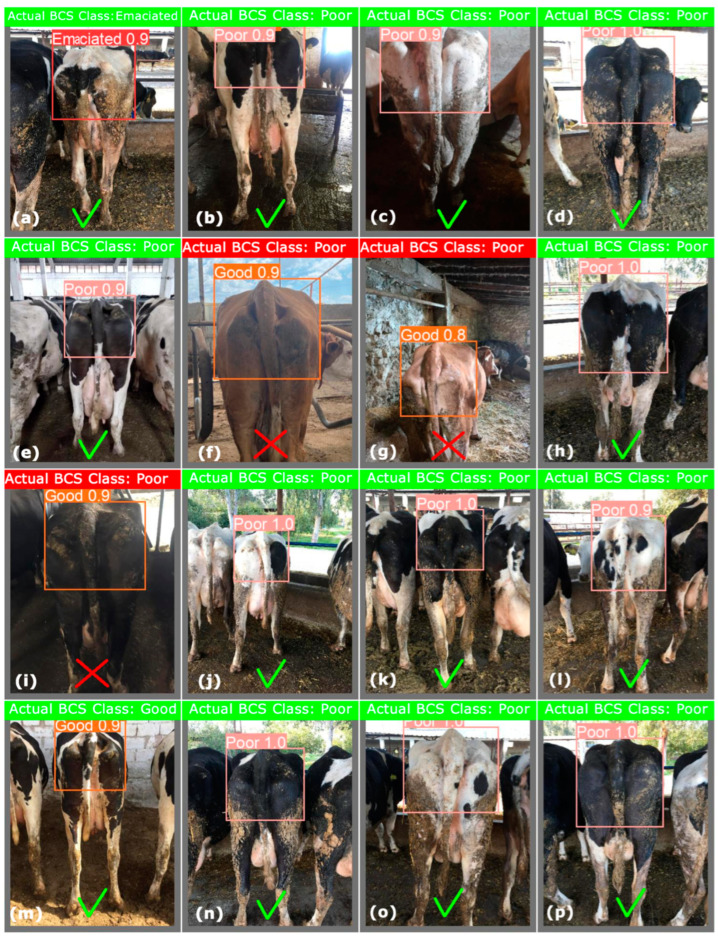
BCS classification results using YOLOv8x in test set. Correct classifications (**a**–**e**,**h**,**j**–**p**) and incorrect classifications (**f**,**g**,**i**).

Further results for the BCS classification of dairy cows in the test subset of the data set that was created in this study are shown in Appendix A and Appendix B. During the creation of the actual class labels by the experts, it was ensured that there was only one cow in each image and only one BCS class label for BCS. However, in the BCS estimations, it can be seen that, in some classifications, such as in Appendix A Figure A1j,m, the result was for two different classes for the same image. In Appendix A Figure A1j, a confidence rate of 0.3 was obtained for the Fat class and 0.7 for the Obese class. In Appendix A Figure A1m, a confidence rate of 0.3 was obtained for the Fat class and 0.4 for the Obese class. In the experimental studies, in case of more than one class prediction for the same image, the classification result with a high mAP value was taken into account for the higher confidence rate of the predicted class. In this case, for both Appendix A Figure A1j,m, the class for BCS was considered as Obese. On the other hand, as shown in Appendix B Figure A2f,g,i, three images with actual BCS class Poor were incorrectly included in the class Good BCS using the proposed YOLOv8x architecture. When analyzing the Poor- and Good-class images in the test set, it can be seen that there were similar BCS images in both sets. Therefore, it was predicted that the incorrect classification for these patterns was due to this reason. In such cases, it may be appropriate to evaluate other parameters for more accurate BCS classification.

The proposed BCS classification system can be a valuable tool for farmers and production decision-makers. It enables the continuous monitoring of cow body condition without the need for expert evaluation, which can be time-consuming and subjective. This automation allows for more consistent and objective assessments, improving herd management decisions. For instance, during early lactation, when cows are most susceptible to negative energy balance, the system can help to identify individuals at risk and guide dietary adjustments to prevent excessive weight loss. Similarly, it can aid in maintaining optimal body condition throughout the production cycle, enhancing overall herd health and productivity. A significant strength of this study lies in the creation of a diverse and well-annotated dataset representing different BCS classes and farm environments. Utilizing expert knowledge for image annotation and BCS classification ensured the accuracy of ground truth labels. Additionally, employing PRCs provided valuable insights into the model’s performance beyond just overall accuracy, especially for imbalanced class distributions.

On dairy farms, an animal that is too thin or too fat is undesirable and can cause many problems. Thin cows are more prone to metabolic disorders, such as ketosis and fatty liver disease. Low body condition can compromise the immune system, making cows more susceptible to infections and diseases such as mastitis. In addition, thin cows often have irregular estrous cycles and lower conception rates. Cows with low body condition scores lack the energy reserves to maintain high milk production. This can lead to a significant reduction in milk yield and quality. Severely underconditioned cows have a higher risk of mortality due to complications associated with metabolic and infectious diseases. On the other hand, overconditioned cows have a higher risk of developing metabolic disorders, such as fatty liver disease and abomasal displacement. In addition, high body condition scores can lead to increased weight on the limbs, causing lameness and other hoof problems. This can have a significant impact on mobility and overall welfare. In addition, fat cows may have a delayed peak in milk production. Excess body fat can hinder the mobilization of energy needed for early lactation, reducing overall milk yield. Maintaining overconditioned cows can lead to higher feed costs, as these cows may require special diets to manage their weight and health. Therefore, the proposed BCS classification system can help to mitigate these problems by enabling early detection and intervention. Farmers can identify under- or over-conditioned cows at an early stage and take preventive action to avoid serious health problems by accurately classifying cows’ body condition scores. In addition, milk production and reproductive performance are improved, contributing to overall farm productivity and profitability, by maintaining cows at optimal body condition scores. Timely intervention based on accurate BCS scoring can help in adjusting diets and management practices to maintain optimal body condition, reducing the risk of metabolic and other health problems.

In this study, the studies [12,13,20,36,37,38,39,45] that presented an automated determination of BCS in dairy cows similar to the proposed classification system for BCS determination were compared in terms of year of publication, number of farms, breed of cows, total number of images in the dataset, proposed methodology, and accuracy metric, as shown in Table 8. In the previously proposed studies, similar to this study, study-specific datasets were created for each study when calculating the accuracy metric. Therefore, there is no publicly available dataset for BCS classification in dairy cows to ensure experimental standardization among the studies. Therefore, this study and the previously proposed studies were only evaluated in terms of the accuracy metric for performance comparison. In addition, human error intervals were also evaluated to measure the precision of the methods in some previously proposed studies [13,20,36,37,38,45]. From Table 5, it can be seen that the proposed study performed better in terms of accuracy in classifying actual and predicted BCS values and obtained significant results. However, it is important to emphasize that the hyperparameter optimization in the proposed YOLOV8x method improves the results and shows a high predictive capacity. Moreover, the proposed study stands out more in terms of cow breeds of Holstein and Simmental, number of farms, and number of images used in the experimental validations. The average BCS classification accuracy for Holstein cows was 82.0%, while for Simmental cows, it was 80.0%. On the other hand, the average accuracy for both cow breeds was 81.0%. When the BCS classification results of Simmental and Holstein cows were evaluated together, higher classification accuracy was obtained for the BCS classification of Holstein cows.

This study has some limitations that need to be acknowledged. The generalizability of the model to other cow breeds and geographical locations might require further investigation. Additionally, the model faced challenges in differentiating cows with similar BCS scores, suggesting the need for further exploration of data augmentation techniques or alternative deep learning architectures specifically designed for fine-tuned image classification tasks. In addition, two different cow breeds, such as Holstein and Simmental, were classified for BCS determination, but it may be important to increase the number of Simmental cows for each BCS class, especially in the dataset, to validate the results.

## 5. Conclusions

This study successfully developed an automated system for classifying BCS in dairy cows into five categories: Emaciated (BCS = 2.0), Poor (BCS = 2.5), Good (BCS = 3.5), Fat (BCS = 4.0), and Obese (BCS = 4.5) using a single-stage YOLOv8x deep learning architecture. The investigation yielded promising results, highlighting the potential of this approach for on-farm BCS assessment. The results of the experimental analyses in the study show that the proposed BCS classification system can easily misclassify Fat with Good classes due to the similar appearance of dairy cows, as in Good and Poor classes. In the BCS classification of Holstein and Simmental breeds separately, higher accuracy was achieved in the BCS classification of Simmental breeds. The study has demonstrated that using the proposed method can decrease the error rate associated with experts’ visual and manual evaluation when determining the BCS of dairy cows. This suggests that the proposed classification system enhances the accuracy and consistency of BCS assessments compared with traditional expert evaluations. In addition, the automated BCS classification system in dairy cows has the potential to provide valuable support to production decision-makers during early lactation to mitigate the effects of negative energy balance.

Currently, the BCS classification system relies on a single image of the cow’s back for BCS determination. To enhance the robustness of the system, it would be beneficial to incorporate additional images and evaluate other relevant parameters. For example, considering multiple images captured from different angles and perspectives would provide a more comprehensive representation of the cow’s body condition. In addition, capturing images of the cow’s back, flanks, and potentially the front may be useful in BCS grouping to obtain a holistic view of its body composition. Moreover, utilizing video sequences to capture dynamic information about cow movement and posture can also provide additional clues for BCS assessment. Future research can focus on developing a real-time BCS determination system capable of analyzing images from multiple angles, thereby enhancing its practical utility in dairy herd management. In addition, integrating the proposed model into a mobile application would allow farmers to easily assess the BCS of their cows on the farm.

## Figures and Tables

**Table 1:** The infrastructure and hardware specifications of the computer used for the experimental analysis.

Hardware	Specification
Computer	Desktop
Central processor (CPU)	4-Core AMD Ryzen3 2200 G 3.5 GHz processor (AMD Ryzen Santa Clara, CA, USA)
Memory (RAM)	32 GB
Mainboard	Gigabyte B450M S2H
Graphical processing unit (GPU)	NVIDIA GeForce RTX 3060 12 GB (NVIDIA, Santa Clara, CA, USA)
Hard disc driver	120 GB (500 MB/s Read/Write) SDD

**Table 2:** Optimization of hyperparameters used in the training of the YOLOv8x network.

Parameters	Value
Weights	YOLOv8x
Epoch	300
Batch size	16
Image size	512
Optimizer	SGD
Mask ratio	4
IoU	0.5
Learning rate	0.01
Momentum	0.937
Mosaic	1.0
Dropout	0.0
Weight decay	0.0005

**Table 3:** Number of images from the training and test sets for each class in the dataset according to BCS class.

BCS Description	BCS Scale	Training Images		Test Images		Total Images		Total
		Simmental	Holstein	Simmental	Holstein	Simmental	Holstein	
Emaciated	2.0	1	71	1	7	2	78	80
Poor	2.5	13	212	3	22	16	234	250
Good	3.5	82	363	11	38	93	401	494
Fat	4.0	156	163	18	17	174	180	354
Obese	4.5	54	29	6	3	60	32	92
	Total	306	838	39	87	345	925	1270

**Table 4:** The confusion matrix resulting from the classification of 126 images for 5 different BCS classes in the test set with the proposed YOLOv8x deep learning architecture.

	**Total**	8	25	49	35	9	126
**Predicted**	**Emaciated**	8					8
	**Poor**		17	1			18
	**Good**		7	44	8	1	60
	**Fat**		1	4	27	2	34
	**Obese**					6	6
		**Emaciated**	**Poor**	**Good**	**Fat**	**Obese**	**Total**
	**Actual**						

**Table 5:** Accuracy scores achieved with YOLOv8x on the basis of images in the test set for each class.

	**Total**	1.00	1.00	1.00	1.00	1.00	5.00
**Predicted**	**Emaciated**	1.00					1.00
	**Poor**		0.68	0.02			0.70
	**Good**		0.28	0.90	0.23	0.11	1.52
	**Fat**		0.04	0.08	0.77	0.22	1.11
	**Obese**					0.67	0.67
		**Emaciated**	**Poor**	**Good**	**Fat**	**Obese**	**Total**
	**Actual**						

**Table 6:** The confusion matrix for classification result in each BCS class for a total of 39 Simmental cow images in the test set.

	**Total**	1	3	11	18	6	39
**Predicted**	**Emaciated**	1					1
	**Poor**		1				1
	**Good**		2	8	2		12
	**Fat**			3	16	1	20
	**Obese**					5	5
		**Emaciated**	**Poor**	**Good**	**Fat**	**Obese**	**Total**
	**Actual**						

**Table 7:** The confusion matrix for classification result in each BCS class for a total of 87 Holstein cow images in the test set.

	**Total**	7	22	38	17	3	87
**Predicted**	**Emaciated**	7					7
	**Poor**		16	1			17
	**Good**		5	36	6	1	48
	**Fat**		1	1	11	1	14
	**Obese**					1	1
		**Emaciated**	**Poor**	**Good**	**Fat**	**Obese**	**Total**
	**Actual**						

**Table 8:** Comparison of previous studies presented in this study for automatic determination of BCS in dairy cows, similar to the proposed classification system for BCS determination.

Study	Year	Number of Dairy Farms	Cow Breed	Number of Images	Methodology	Accuracy (%)
Bercovich et al. [20]	2013	1	Holstein	151	Body shape signature and Fourier descriptors	72.0 (with 0.5 unit error range)
Alvarez et al. [36]	2018	3	Holstein	1661	CNN	78.0 (with 0.25 unit error range)
Li et al. [38]	2019	2	Holstein	2231	YOLOv2 and ResNet50	78.15 (with 0.25 unit error range)
Alvarez et al. [37]	2019	N/A	N/A	1661	CNN and ensemble Techniques	82.0 (with 0.25 unit error range)
Çevik and Boğa [12]	2019	N/A	N/A	184	VGG19 and R-CNN	67.39
Yukun et al. [45]	2019	1	Holstein-Friesian	686	CNN-based deep learning model	77.0 (with 0.25 unit error range)
Liu et al. [13]	2020	1	N/A	295	Ensemble learning model based on bagging and boosting	76.0 (with 0.25 unit error range)
Çevik [39]	2020	10	Holstein	505	Pre-trained VGG19-bsaed CNN (mobile app.)	78.0
Ours	2024	20	Simmental and Holstein	1270	YOLOv8x-based deep learning architecture	80.0 (Simmental) 82.0 (Holstein) 81.0 (Average)

N/A: not applicable.

## Data Availability

The raw data supporting the conclusions of this article will be made available by the authors on request.
